# The Physician’s Prescription Book

**Published:** 1857-01

**Authors:** 


*The Physician's Prescription Book: Containing List of Terms, Phrases, Contractions, and Abbreviations used in Prescriptions, with Explanatory Notes; also, the Grammatical Construction of Prescriptions, &c. To which is added a Key, Containing the Prescriptions in an Unabreviated Form, with a Literal Translation, intended for the Use of Medical and Pharmaceutical Students.* By Jonathan Pereira, M.D. F.R.S. Second American, from the Twelfth London Edition. Philadelphia, Lindsay & Blakiston, 1857.

This is a small duodecimo volume, of two hundred and eighty-two pages, neatly printed and bound in cloth. Its design and contents are sufficiently indicated by the foregoing copious title page. By the medical student, the apothecary, and indeed all who either write or fill prescriptions, it will be found very convenient and useful.

